# Identification and Quantitation of the Bioactive Components in Wasted *Aralia elata* Leaves Extract with Endothelial Protective Activity

**DOI:** 10.3390/molecules28155907

**Published:** 2023-08-06

**Authors:** Dan Gao, Chong-Woon Cho, Zemin Yang, Xiwen Li, Jong-Seong Kang

**Affiliations:** 1 Institute of Chinese Materia Medica, China Academy of Chinese Medical Sciences, Beijing 100700, China; dgao@icmm.ac.cn (D.G.); yangzm0615@163.com (Z.Y.); 2 College of Pharmacy, Chungnam National University, Daejeon 34134, Republic of Korea; chongw113@naver.com; 3 College of Traditional Chinese Medicine, Yunnan University of Chinese Medicine, Kunming 650500, China

**Keywords:** biological activities, HPLC–PDA–ESI–MS/MS, *Aralia elata* (Miq.) Seem, multivariate data analysis, endothelial cell dysfunction

## Abstract

*Aralia elata*, a renowned medicinal plant with a rich history in traditional medicine, has gained attention for its potential therapeutic applications. However, the leaves of this plant have been largely overlooked and discarded due to limited knowledge of their biological activity and chemical composition. To bridge this gap, a comprehensive study was conducted to explore the therapeutic potential of the 70% ethanol extract derived from *Aralia elata* leaves (LAE) for the treatment of cardiovascular disease (CVD). Initially, the cytotoxic effects of LAE on human umbilical vein endothelial cells (HUVECs) were assessed, revealing no toxicity within concentrations up to 5 μg/mL. This suggests that LAE could serve as a safe raw material for the development of health supplements and drugs aimed at promoting cardiovascular well-being. Furthermore, the study found that LAE extract demonstrated anti-inflammatory properties in HUVECs by modulating the PI3K/Akt and MAPK signaling pathways. These findings are particularly significant as inflammation plays a crucial role in the progression of CVD. Moreover, LAE extract exhibited the ability to suppress the expression of adhesion molecules VCAM-1 and ICAM-1, which are pivotal in leukocyte migration to inflamed blood vessels observed in various pathological conditions. In conjunction with the investigation on therapeutic potential, the study also established an optimal HPLC–PDA–ESI–MS/MS method to identify and confirm the chemical constituents present in 24 samples collected from distinct regions in South Korea. Tentative identification revealed the presence of 14 saponins and nine phenolic compounds, while further analysis using PCA and PLS-DA allowed for the differentiation of samples based on their geographical origins. Notably, specific compounds such as chlorogenic acid, isochlorogenic acid A, and quercitrin emerged as marker compounds responsible for distinguishing samples from different regions. Overall, by unraveling its endothelial protective activity and identifying key chemical constituents, this research not only offers valuable insights for the development of novel treatments but also underscores the importance of utilizing and preserving natural resources efficiently.

## 1. Introduction

Cardiovascular diseases (CVDs) can be caused by high blood pressure, atherosclerosis, easy blood clotting, and heart enlargement, which constitute the leading health problem all over the world [1]. The healthy vascular endothelium is beneficial to the prevention of cardiovascular diseases. Healthy endothelium has vasodilator, anti-atherosclerosis, and anti-inflammatory properties [2]. Unfortunately, some risk factors for cardiovascular disease can lead to endothelial cell dysfunction, which is a key event in the pathogenesis of atherosclerosis, coronary artery contraction, and myocardial ischemia [3,4]. Interestingly, endothelial cell dysfunction is a reversible phenomenon, which provides new ideas for CVD treatment based on its reversal [5].

Recently, there is growing recognition that inflammation plays a crucial role in the development and progression of atherosclerosis, and numerous inflammatory markers and cells have been identified as key contributors to the process [6,7]. Among these markers, C-reactive protein (CRP), monocyte chemoattractant protein-1 (MCP-1), intercellular adhesion molecule-1 (ICAM-1), and vascular cell adhesion protein 1 (VCAM-1) have all been implicated in endothelial dysfunction and the development of vascular disease [8,9]. In particular, elevated levels of CRP have been identified as a risk factor for vascular disease, and chronic exposure to inflammatory stimuli or injury can lead to oxidative stress, endothelial dysfunction, and intimal hyperplasia through an impact on nitric oxide bioavailability [10,11]. Recent research has also pointed to the importance of several signaling pathways in the development of cardiovascular disease. The extracellular signal-regulated kinase (ERK)1/2 and p38 mitogen-activated protein kinase (p38-MAPK) pathways are known to play a key role in cardiac fibroblast proliferation, and the dysregulation of these pathways has been linked to arrhythmias, myocardial infarction, hypertension, and heart failure. Conversely, the PI3K-Akt signaling pathway promotes metabolism, proliferation, cell survival, growth, and angiogenesis in response to extracellular signals [12,13,14]. Another pathway that has been implicated in the pathogenesis of myocardial ischemia is the Janus kinase/signal transducer and activator of transcription (JAK/STAT) pathway [15]. Interference with or inactivation of this pathway has been shown to improve recovery in cardiac function [16]. Given the critical role of these pathways in cardiovascular disease, the evaluation of specific materials’ effects on the regulation of these pathways could be an effective means of identifying potential compounds or extracts for use in the development of new treatments for cardiovascular disease.

*Aralia elata* (Miq.) Seem is a widely consumed edible vegetable in various regions including Korea, China, Far East Russia, and Japan [17]. The plant has a long history of use in traditional folk medicine where it has been reported to possess a variety of beneficial properties [18]. The bark and roots have been used for the treatment of conditions such as neurasthenia, rheumatoid arthritis, diabetes mellitus, gastrospasm, constipation, and hepatitis [19,20,21]. These benefits have been attributed to the plant’s different biological activities, including cytotoxic, anti-inflammatory, liver-protecting, antioxidative, antiviral, and anti-diabetic properties [22,23,24]. Studies on the plant have also identified triterpenoid saponins as the main active components [24,25]. The saponins in Aralia species, specifically Aralosides, are similar in effect to panaxosides, the active ingredients found in ginseng [26]. These saponins contribute to Aralia’s ability to increase energy, strengthen the body, and improve the body’s hypoxia ability in regard to the cardiovascular system and other parameters [27]. Despite its numerous medicinal uses, the leaves of *A. elata* (LAE) are often treated as waste and discarded. This results in a significant waste of resources. Therefore, it is of utmost importance to identify the chemical composition and evaluate the activity of the LAE for its potential reutilization.

The aim of this study was to investigate the potential of LAE extracts to regulate the expression levels of ICAM-1, VCAM-1, and inflammatory processes in LPS-stimulated human umbilical vein endothelial cells (HUVECs). To identify the bioactive components of LAE, an integrated analytical strategy using high-resolution and high-selectivity HPLC methods with diode array detection (HPLC–DAD) and HPLC–MS was established. In addition, a simple HPLC–DAD and HPLC–evaporative light scattering detection (ELSD) method was developed for the relatively quantitative analysis of bioactive components found in LAE from different cities and seasons. This approach enabled the identification of the chemical constituents and biological activities of LAE and contributed to the growing body of knowledge on the endothelial protective activity of LAE.

## 2. Results and Discussion

### 2.1. Cytotoxic Effects of LAE Extract in HUVECs

**Figure 1:**
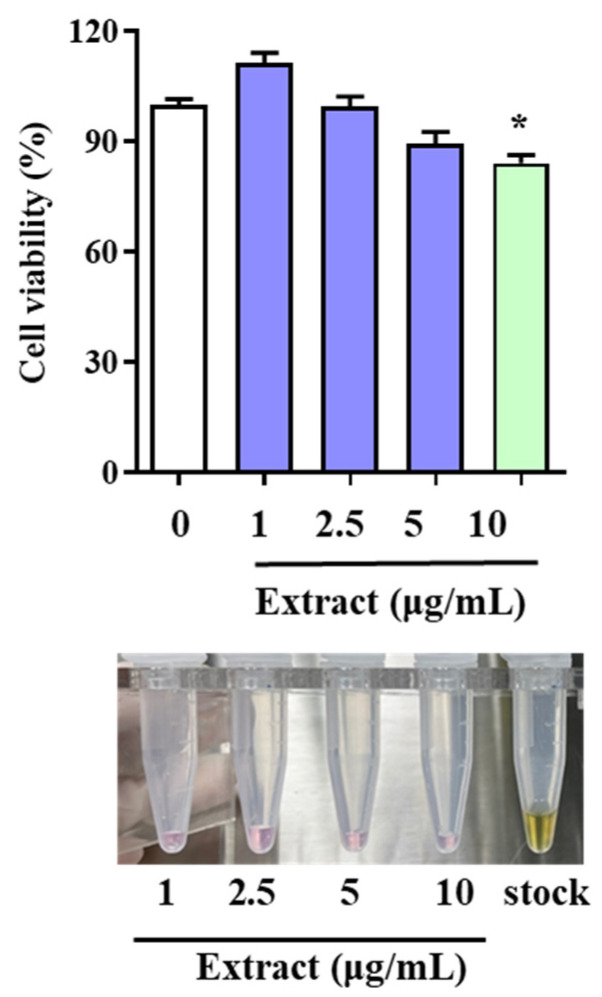
Effects of LAE extract on HUVEC viability. Cell viability was determined using MTT assay, and optical densities at 565 nm are shown. Data are expressed as the means ± SDs. * *p* < 0.05 vs. not-treated group.

To determine whether the LAE extract has cytotoxic effects, HUVECs were treated with varying concentrations of the extract (1, 2.5, 5, and 10 μg/mL) for 48 h, and cell viability was evaluated. The results of the study revealed that the LAE extract had no toxicity to the HUVECs even at concentrations as high as 5 μg/mL, as the cell viability remained above 90%, indicating that the extract did not have any negative impact on the cells (Figure 1). However, at a concentration of 10 μg/mL, the cell viability significantly decreased compared to the non-treated group, suggesting that the extract might have a toxic effect on HUVECs at higher concentrations.

Despite this observation, the study highlighted the satisfactory activity of the LAE extract, even at a low concentration. This finding is significant as it suggests that the extract has potential therapeutic benefits for different conditions, such as cardiovascular diseases, without harming non-target cells or tissues. This suggests that LAE can serve as a raw material for health supplements or even drugs to protect cardiovascular health. Utilizing discarded LAE as a treatment material for CVD not only helps to reduce waste and protect the environment, but also provides a cost-effective and sustainable source of medicinal plant extracts. 

### 2.2. LAE Extract Attenuates Inflammatory Signaling Pathways in HUVECs

**Figure 2:**
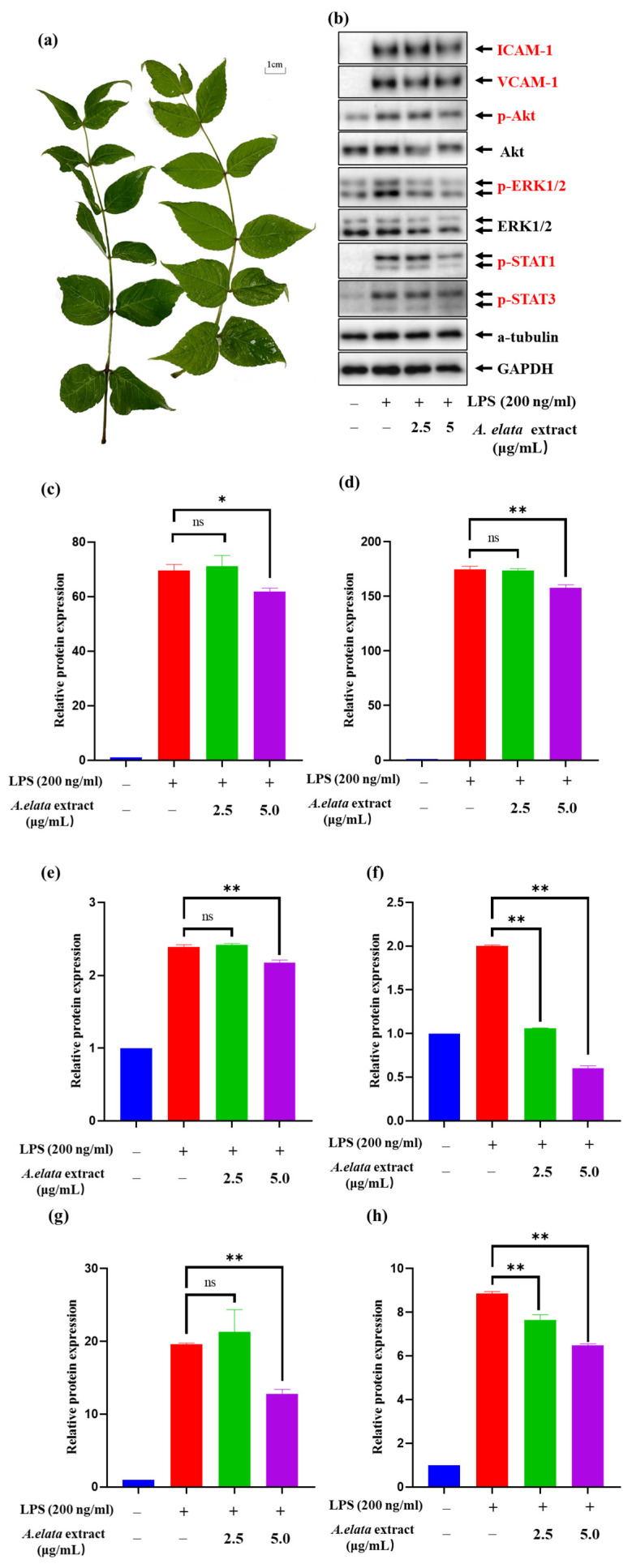
The picture of (**a**) LAE and the effects of LAE extract on LPS-induced HUVEC activation and expression of (**b**) different inflammatory factors; the relative protein expression of (**c**) ICAM-1, (**d**) VCAM-1, (**e**) p-Akt, (**f**) p-ERK1/2, (**g**) p-STAT1, and (**h**) p-STAT3. The experiment was repeated three times and similar results were acquired. Data are expressed as the means ± SDs. ns: not significant, * *p* < 0.05 and ** *p* < 0.01 vs. the LPS-treated group.

To further understand the cardio-protective effect of LAE extract and to assess the potential signaling pathways corresponding to the characters of this extract, the expression of adhesion molecules VCAM-1 and ICAM-1, MAPK/ERK pathway-related protein p-ERK1/2, P13K/AKT pathway-related protein p-AKT and JAK/STAT related protein p-STAT1 and p-STAT3 were analyzed. The picture of LAE was shown in Figure 2a. Figure 2b demonstrated that LPS stimulation induces a significant increase in the expression of those related proteins and LAE extract could play a vital role in cardiovascular disease through regulation of the adhesion molecules, inflammation signaling pathway of MAPK/ERK, P13K/AKT, and JAK/STAT.

Figure 2c,d demonstrated that the treatment of LAE extract at a concentration of 5 μg/mL could significantly reduce the ICAM-1 and VCAM-1 levels compared with the control group (*p* < 0.05). The study suggests that LAE extract can significantly suppress the expression of VCAM-1 and ICAM-1, important adhesion molecules that play a crucial role in leukocyte migration into inflamed vessels. The expression of both VCAM-1 and ICAM-1 is elevated in various pathological states, including atherosclerosis, endothelial dysfunction, and chronic inflammation. The ability of LAE extract to reduce VCAM-1 and ICAM-1 expression may be an essential mechanism for the extract’s anti-inflammatory effects. Several studies have reported the activation of the PI3K/Akt pathway and its contribution to the upregulated production of inflammatory cytokines in activated endothelial cells [28,29]. Consequently, the effects of LAE extract on LPS-induced PI3K/Akt activation in HUVECs were analyzed. As shown in Figure 2e, LAE extract abrogated the LPS-induced preserved activation of the PI3K/Akt signaling following the LPS challenge. LAE extract, even at a concentration of 5 μg/mL, also significantly decreased the levels of p-Akt compared with LPS-treated controls. Taken together, our data suggest that the cardiac protection by LAE extract against LPS challenge was mediated, at least in part, by an attenuation of PI3K/Akt signaling suppression. 

Next, the effects of LAE extract on the Erk1/2 MAPK signaling pathway were examined, which is involved in the regulation of downstream pro-inflammatory expression in HUVECs. The results in Figure 2f showed that LPS (200 ng/mL) treatment for 17 h induced a rapid increase in p-Erk1/2 protein levels in HUVECs. Co-treatment with LAE extract at concentrations of 2.5 and 5 μg/mL could reduce the level of p-Erk1/2 by 94.1% and 140.0% to that of the LPS-only group, respectively, which suggest that LAE extract could improve LPS-induced inflammation via inhibiting the MAPKs signaling pathways.

According to the previous study, STAT1/3 are major signaling molecules that regulate a variety of inflammatory activities [30]. Normal STAT signaling induced in response to cytokines generates multiple signaling pathways along with the signal cross-talk, for example, certain STATs mediate multiple kinase signals, including MAPKs and MAPK kinase (MKK)-dependent serine kinase [31]. Our Western blotting data show that the pretreatment of 2.5 and 5 μg/mL of LAE extract can decrease the phosphorylation of both STAT1 and STAT3 (Figure 2g,h), respectively, which suggest that LAE extract could regulate the JAK/STAT signaling pathway to treat the cardiovascular diseases. These results reveal that LAE extract could be a promising natural treatment for CVD and reinforce the importance of exploring natural resources for their beneficial effects on human health. However, further studies are needed to elucidate the underlying mechanisms of the extract’s effects on CVD and to validate its clinical efficacy.

### 2.3. Chemical Profiling of Triterpenoid Saponins in LAE Extract

To profile the major constituents of LAE, the optimal HPLC–PDA–ESI–MS/MS method was performed to receive the valuable chemical properties of the main constituents. The chemical structure of each component was identified and confirmed by the following described progress: first, the UV spectra of the peaks were acquired in scan mode (200–400 nm) of HPLC–PDA, which could be very useful for providing the information for discriminating the chemical structure type. Second, the precursor ions provided the important signal to acquire the accurate molecular weights and authentic molecular formulas of those active components. Thirdly, the fragmentation ions afford a more detailed structural information of the beneficial components.

**Figure 3:**
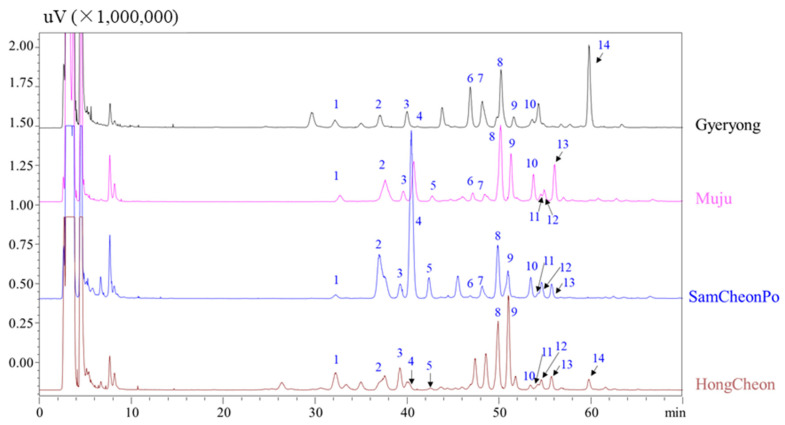
HPLC–ELSD chromatograms of LAE extracts harvested from different cities. The information of peaks 1–14 are listed in Table 1.

Figure 3 displays the typical HPLC–ELSD chromatograms of LAE obtained from Gyeryong, Muju, SamCheonPo, and Hongcheon. All peaks had a maximum UV absorbance near 200 nm, which matched with the properties of triterpenoids saponins. In MS spectra, [M + Na]^+^, [M + H]^+^, and/or [M + NH_4_]^+^ in positive mode as well as [M − H]^−^ or/and M + HCOOH − H]^−^ in negative mode, both provided the clearly molecular weight information for the active components. The aglycone and connected resides could be identified by fragmentation ions [32]. Thus, based on the literature and ESI–MS/MS, peak 1 was determined as congmuyenoside III; peak 2, congmuyenoside G; peak 4, ecalbasaponin III; peak 5, quinoasaponin 2; peak 7, congmuyenoside IX; peak 8, congmuyenoside X, peak 9, congmuyenoside V; peak 10, Araloside G; and peak 11, silphioside. The retention time, precursor ions in positive as well as negative modes, and chemical names are summarized in Table 1. 

The identification of these specific triterpenoid saponins provides insights into the underlying chemical constituents of LAE extract that may contribute to its anti-inflammatory effects. Previous studies have suggested that triterpenoid saponins, such as those identified in this study, have anti-inflammatory, antioxidant, and hepatoprotective activities [33]. For instance, congmuyenoside III has been shown to have anti-inflammatory effects by suppressing the activation of the NF-κB pathway [34]. Congmuyenoside G exhibited antitumor activity and induced apoptosis in human cancer cells [35]. Ecalbasaponin III demonstrated protective effects against liver injury induced by CCl4 [36]. Quinoasaponin 2 showed antioxidant and hepatoprotective effects in a mouse model of liver injury [37]. Congmuyenoside X exerted hepatoprotective effects in a mouse model of liver injury by inhibiting the activation of the NF-κB signaling pathway [38]. These findings suggest that the identified triterpenoid saponins may be key contributors to the anti-inflammatory effects of LAE extract.

**Table 1:** Identification of triterpenoid saponins in LAE by HPLC–PDA–ESI–MS/MS.

No.	RT ^a^ (min)	Positive Mode (*m*/*z*)	Negative Mode (*m*/*z*)	MW ^b^	Trivial Name	MS^2^, Ions *m*/*z* (Relative Abundance, %)	Reference
1	32.13	1300.80 [M+ NH_4_]^+^	1327.75 [M + HCOOH − H]^−^	1282.8	Congmuyenoside III	671.2 (25), 455.3 (75)	[39]
2	36.57	1305.85 [M + Na]^+^	1327.90 [M + HCOOH − H]^−^	1282.9	Congmuyenoside G	691.2 (30), 455.3 (70)	[39]
3	39.42	981.60 [M + Na]^+^	1003.65 [M + HCOOH − H]^−^	958.7	unknown	509.2 (15), 471.3 (85)	-
4	40.28	981.70 [M + Na]^+^	1003.70 [M + HCOOH − H]^−^	958.7	Ecalbasaponin III	673.4 (70), 493.3 (30)	[40]
5	42.16	981.70 [M + Na]^+^	1003.70 [M + HCOOH − H]^−^	958.7	Quinoasaponin 2	819.5 (65), 455.3 (35)	[36]
6	47.73	819.60 [M + NH_4_]^+^	841.70 [M + HCOOH − H]^−^	796.6	unknown	641.4 (85), 439.4 (15)	-
7	48.22	1108.75 [M + NH_4_]^+^	1089.23 [M − H]^−^	1090.7	Congmuyenoside IX	1143.4 (80), 455.3 (20)	[41]
8	50.21	1284.90 [M + NH_4_]^+^	1266.10 [M − H]^−^	1266.9	Congmuyenoside X	1127.6 (85), 455.3 (15)	[41]
9	51.09	1122.70 [M + NH_4_]^+^	1149.85 [M + HCOOH − H]^−^	1104.9	Aralia Saponin V	981.5 (70), 455.4 (30)	[42]
10	53.68	1122.70 [M + NH_4_]^+^	1103.90 [M + HCOOH − H]^−^	1104.8	Araloside G	997.48 (60), 455.4 (40)	[43]
11	54.08	965.60 [M + Na]^+^	987.55 [M + HCOOH − H]^−^	942.6	Silphioside	803.5 (65), 439.4 (35)	[43]
12	54.52	960.65 [M + NH_4_]^+^	987.55 [M + HCOOH − H]^−^	942.6	unknown	641.4 (70, 439.4 (30)	-
13	57.72	960.65 [M + NH_4_]^+^	987.55 [M + HCOOH − H]^−^	942.6	unknown	657.4 (80), 455.4 (20)	-
14	59.76	817.60 [M + NH_4_]^+^	793.75 [M − H]^−^	794.8	unknown	643.4 (75), 455.4 (25)	-

Note: ^a^ RT: retention time; ^b^ MW: molecular weight.

However, further studies are needed to investigate the potential synergistic effects that exist between these triterpenoid saponins and other constituents present in LAE extract. Additionally, the bioavailability and pharmacokinetics of these constituents need to be further elucidated to better understand their therapeutic potential. Nonetheless, the identification of the specific triterpenoid saponins in LAE extract may pave the way for the development of novel, natural anti-inflammatory agents for the treatment of various inflammatory disorders. 

### 2.4. Changes in the Chemical Constituents in the Leaves of A. elata from Different Seasons and Cities

It is known that there are large differences in the chemical constitutions of natural products depending on the harvest seasons and growth environments, and these differences could cause different or even opposite efficacy [44,45]. Therefore, it is necessary to determine the appropriate harvest season and cultivated city by comparing the content differences between chemical components. Previous studies demonstrated that the LAE in spring has comparatively fewer triterpenoid saponins, while those active saponins showed more in samples collected from autumn [46]. 

In the present study, the representative flavonoids and phenolic acid compounds in the LAE were evaluated, and the results are shown in Figure S1. Firstly, the LAE from the same location in Gyeryong city were collected and analyzed by HPLC; it was found that the amount of peaks 1 to 9 in LAE harvested in spring and summer was very low, and even some peaks were difficult to detect (Figure S1). The reasons for this may be related to a variety of biological and environmental factors [47]. For example, many plants may allocate more energy and resources to growth and developmental constructs than to the accumulation of secondary metabolites such as phenolic acids during the spring and summer growing seasons in order to grow rapidly [47,48]. In addition, in summer, plants may produce more of other protective compounds, such as soluble sugars and lipids, that are more beneficial to plants against excessive temperatures or drought conditions than phenolic acids [49,50]. And this phenomenon has also been documented in other species, such as figs, *Camellia sinensis*, and Arabian Lilac (*Vitex trifolia* var. *Purpurea*) [51,52,53]. On the contrary, the content of active ingredients in the LAE harvested in autumn is very high, especially chlorogenic acid (peak 1). This is due to several interrelated factors associated with the natural life cycle of many plants and their response to environmental stressors. Chlorogenic acid (CGA) has been identified as a critical factor in combating oxidative stress in plants. In the autumn, plants experience various stressors such as lower temperatures, a reduced light intensity, and altered photoperiods, all of which can lead to the production of reactive oxygen species (ROS) [54,55]. Higher levels of chlorogenic acid help mitigate the damaging effects of these reactive molecules. Additionally, chlorogenic acid plays a significant role in photosynthesis. Under conditions of a reduced photosynthetic activity, such as the shorter daylight hours typically observed in the fall, there tends to be an increase in CGA synthesis and accumulation, which is believed to be a compensatory adaptation [56,57]. Moreover, autumn often sees an increase in pathogen attacks, and CGA is a noteworthy compound that plants employ to defend themselves against microbial pathogens and pests. As a result, its production is naturally enhanced during periods of heightened risk [58]. CGA also plays a crucial role in regulating plant growth and the onset of senescence. According to the previous report, the levels of this compound tend to rise during the later stages of leaf development, which aligns with the fall season. In an adaptive response to environmental changes during autumn, plants may produce more CGA in preparation for harsh winter conditions [59]. Therefore, the result suggested that the best harvest season for LAE was autumn, which is similar to the previous analysis results of saponins in LAE [46]. 

Secondly, the LAE harvest from Gyeryong, Muju, SamCheonPo, and Hong Cheon were comprehensively analyzed by HPLC–UV and ELSD. Figure 3 shows the distribution of the triterpene saponins in the LAE collected in different cities. From Figure 3, it can be seen that Ecalbasaponin III has the highest content in the samples harvested from SamcheonPo, while peak 14 in Gyeryong was the highest. This may be related to environmental factors in the harvesting area, such as sunlight, water availability, nutrient availability, temperature, and the presence of pests or disease. Triterpene saponins are secondary metabolites typically produced by plants in response to stressful conditions, so areas with more stressors may result in higher yields of triterpene saponins [60]. Additionally, the composition of the soil at the sample site, including nutrient content and pH, can significantly affect the synthesis of triterpene saponins [61,62]. Certain nutrients are necessary for saponin biosynthesis, and their availability can vary from region to region [63]. Aralia Saponin V (peak 9) has antihyperglycemic, hypolipidemic, and antioxidant activities and is the most abundant in the leaves harvested from HongCheon, followed by Muju [64]. Congmuyenoside X is considered a bioactive component in LAE and has roughly the same content in the LAE from the four cities [65]. It is interesting to note that latitude can significantly impact the saponin content in plants. For instance, in *Dioscorea zingiberensis*, the content of steroid saponin varies depending on the latitude of the growing region. This is due to the varying intensity and duration of sunlight, temperature, and other environmental factors that are associated with different latitudes [66]. These factors can impact the biosynthesis of ginsenosides in the plant, which is consistent with the results of our study. These findings suggest that the content and composition of the chemical constituents of LAE extract may influence its therapeutic potential, and the choice of harvest location and season may impact the extract’s bioactivity.

**Figure 4:**
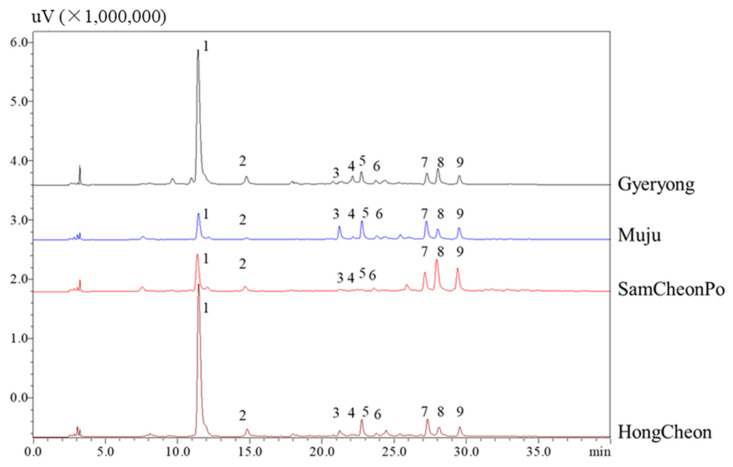
HPLC–UV chromatograms of *A. elata* leaves harvested from different cities. Peak 1: chlorogenic acid; peak 5, rutin; peak 6: hyperoside, peak 7: isochlorogenic acid A; peak 8: quercitrin; peak 9: isochlorogenic C; other peaks were unknown.

Thirdly, to understand the phenolic acid and flavonoid changes from different regions, HPLC–PDA analysis was performed (Figure 4). The structures of these phenolic acids and flavonoids were determined through a comparison with standards according to the previous literature [45]. The results indicate that the content of chlorogenic acid in Gyeryoung and Hongcheon is much greater than that in Muju and Samcheonpo. This may be related to local precipitation, as the synthesis of phenolic acids is closely related to local precipitation. Precipitation can influence the synthesis of phenolic acids in a number of ways. Strong precipitation or rainfall can leach nutrients from the soil, thus affecting plant nutrient availability [67,68]. A reduced nutrient availability has been shown to increase the synthesis of phenolic composites in plants as it stimulates them to produce more secondary metabolites, including phenolic acids. Furthermore, excess water can lead to waterlogging or flooding conditions, which causes a decrease in soil oxygen levels (hypoxia). This condition imposes stress on plants, which can trigger the production of phenolic acids as a response. Certain phenolic compounds are also known to have a role in enhancing plant tolerance to flooding, as they help alleviate oxidative damage caused by hypoxia. On the other hand, a lack of sufficient precipitation can lead to drought stress, which is known to stimulate the production of phenolic acids. Drought stress leads to oxidative stress in plants, and phenolic acids have been associated with an increase in antioxidant activity. Additionally, several studies indicate high phenolic acid content in plants grown in drought conditions [69,70,71]. For example, a previous study demonstrated that drought increased the synthesis of phenolic compounds, including phenolic acids, in olive trees [72]. Ultimately, precipitation, either in excess or deficiency, imposes different kinds of stress on plants, leading to an increase in the synthesis of phenolic acids, a type of secondary metabolite that helps plants adapt to varying environmental conditions. The HPLC profile of Gyeryong is similar to that of Hongcheon, while the profile of Muju and Samcheonpo are similar. The most representative flavonoid, quercitrin, has the highest content in Samcheonpo. Interestingly, quercetin has been considered to be effective in the prevention and treatment of CVD because of its antioxidant, anti-inflammatory, and anti-apoptotic activities, its protective effect on NO, anti-aggregation effect, blood pressure-lowering effects, and beneficial effects on endothelial dysfunction [73,74]. The results of the HPLC analysis above indicated that the contents of triterpenoid saponins, phenolic acids, and flavonoids fluctuated greatly in different LAE samples.

Therefore, to better distinguish the leaves of LAE from different cities, multivariate data analysis, including HCA, PCA, and PLS-D were performed, which can provide a basis for identifying the source and quality of LAE extract based on their chemical compositions. The findings of this study offer important insights into the diversity of chemical constituents present in LAE extract, and it is possible to influence the extract’s bioactivity and therapeutic potential.

### 2.5. HCA Heatmap Analysis

The hierarchically clustered heatmap is a combination of clustergrams and heatmap, both of which are generally hired for multiple data visualization. These two approaches are uncomplicated to describe and can offer the characteristics of the initial data. Heatmaps can directly reflect the changes between different collected samples (such as the content of the chemical composition) according to the changes of color, while clustergrams can display the similarity and correlation between samples. Therefore, in this study, the hierarchically clustered heatmap with complete linkage after Pareto scaling in MetaboAnalyst 5.0 platform was employed to evaluate the similarity and difference of the phenolic and flavonoid component profiles in the LAE from different regions in the original data.

**Figure 5:**
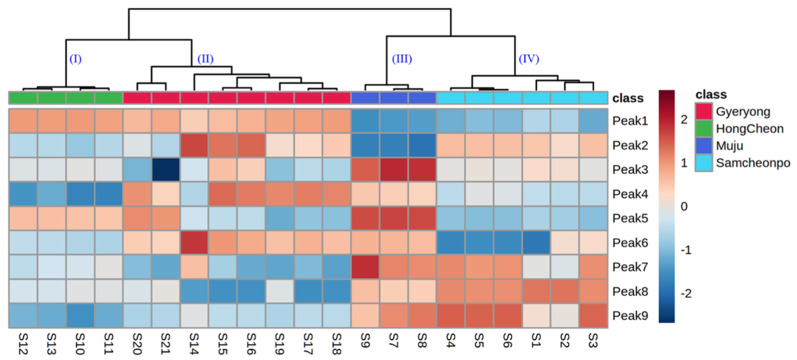
Dendrogram and heatmap of *A. elata* leaves from Gyelong, HongCheon, Muju, and Samcheonpo.

The results (Figure 5) showed that all of the LAE samples exhibited a significant spatial aggregation and could be divided into four groups. The samples from HongCheon city were gathered in Cluster I with the higher contents of chlorogenic acid and rutin; those harvested from Gyeryong were distributed in Cluster II and exhibited a lower content of isochlorogenic acid C and isochlorogenic A; Cluster III mainly included the samples from Muju city with the lowest contents of chlorogenic acid; the other samples, including those from Samcheonpo were all clustered in Cluster IV. These results were in accordance with the geographical position of harvested regions and were highly consistent with the visual comparison of their chromatograms. However, how to more accurately explain the difference between the individual samples in the same group was found to be a complication of HCA. From this point of view, the chemical pattern recognition approaches, including PCA and PLS-DA, should be taken into account for further evaluation of sample clustering based on geographical regions.

### 2.6. Principal Component Analysis

**Figure 6:**
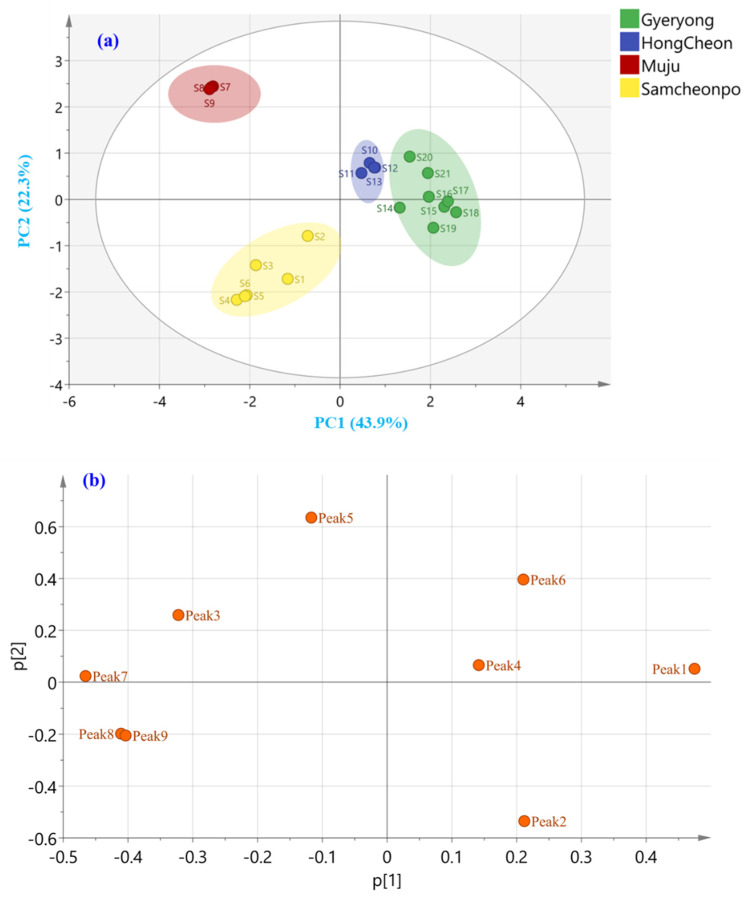
Unsupervised (**a**) PCA score and (**b**) loading plot of *A. elata* leaves harvested from Gyeryong, Hongcheon, Muju, and Samcheonpo.

To evaluate the discrimination ability of the active components and classify the samples from different regions, PCA was performed to find the potential markers. The PCA model resulted in nine principal components (PCs) that could explain 66.1% of the total variance (R^2^X), with a Q^2^ value of 0.767. Then, Hotelling’s T^2^ and distance to the model plots were performed to identify the potential outliers. Hotelling’s T^2^ plot, which measures how far each target is from the model center, did not indicate any outlier using 95% and 99% confidence levels for moderate and strong outliers, respectively (Figure S2a). As the result, the distance to the model plots, which show the deviations between the data and the PCA model, point to any outliers (Figure S2b). Therefore, the PCA model used in this study consisted of 21 samples. As shown in the PCA score plot (Figure 6a), the first two principal components PC1 and PC2 explained 43.9% and 22.3% of the total variance, respectively. Considering the geographical origin, the PCA plot indicated a well-differentiated cluster for LAE samples from different cities. The corresponding diagram obtained by HCA displayed a similar grouping pattern of the samples based on the geographical origin. The loading score scatter plot is displayed in Figure 6b, which demonstrates that peak 1 (chlorogenic acid) and peak 7 (isochlorogenic acid A) have a great potential to discriminate the LAE from different regions. To evaluate the discrimination ability of the active components and classify the samples from different regions, PCA was performed to find the potential markers. PCA model was built considering three replicates per sample, and it showed the variance between the analyzed samples according to their corresponding HPLC–PDA chromatograms.

### 2.7. Partial-Least-Squares Discriminate Analysis

PCA is a widely used tool for identifying patterns and clusters within complex datasets. However, PCA is an unsupervised approach and does not automatically provide information about class membership or the factors that contribute to sample discrimination. Consequently, the PLS-DA model was utilized to look for the potential secondary metabolites that contributed to the sample discrimination based on the investigated geographical origins (Gyeryong, Hongcheon, Muju, and Samcheonpo). The performance and validation of the PLS-DA model were assessed by the R2Y and Q2 values computed by the cross-validation in the SIMCA program by excluding the potential overfitting using permutation tests, and by evaluating the predictive ability of the model through external validation. The correct classification rate (CCR%) of the samples included in the prediction set (containing 20% of the samples) was considered for external model validation.

**Figure 7:**
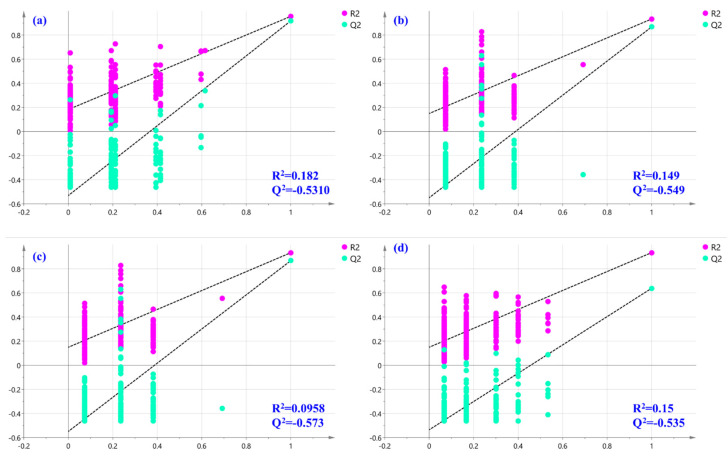
Permutation tests were performed with 200 permutations of Y variables of the PLS-DA model for (**a**) Gyeryong, (**b**) Hongcheon, (**c**) Muju, and (**d**) Samcheonpo.

**Figure 8:**
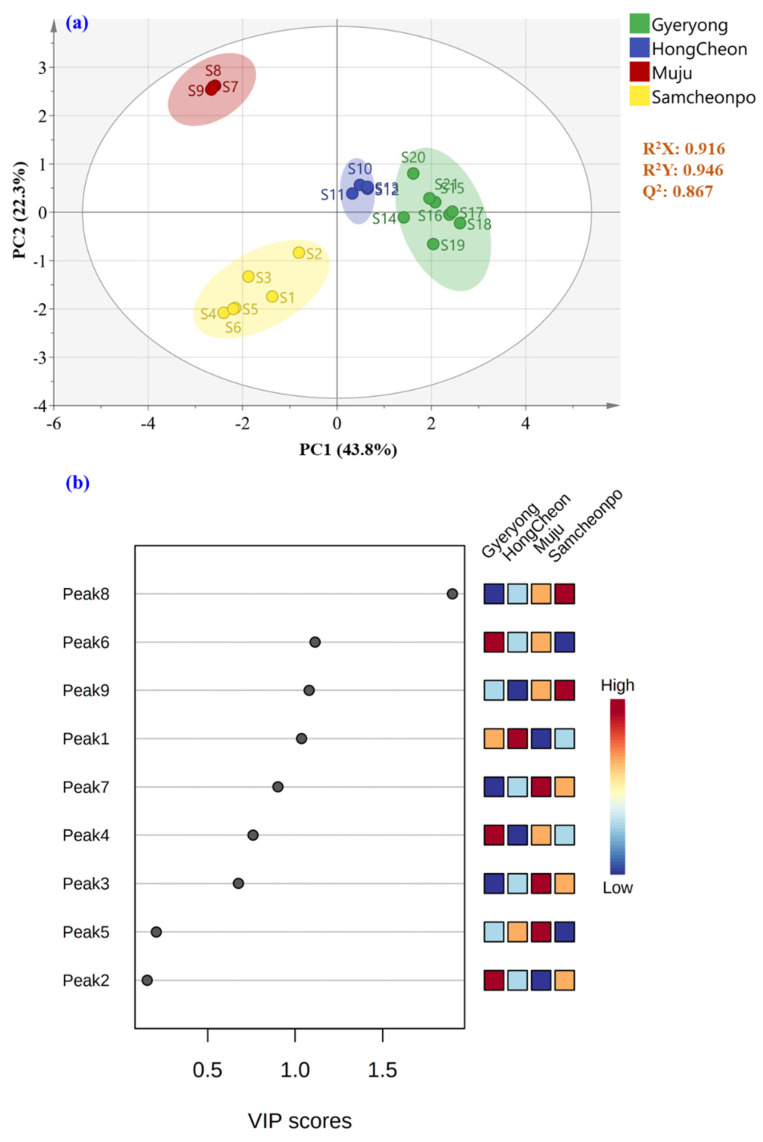
Supervised (**a**) PLS-DA score and (**b**) variable importance in the projection plot of LAE harvested from Gyeryong, Hongcheon, Muju, and Samcheonpo.

An acceptable model explaining 91.6% of the variance was acquired for the categorization of LAE according to the harvested location, with R^2^Y = 0.946, Q^2^ = 0.867, and CV-ANOVA *p*-value < 0.05. Furthermore, a permutations test (200 permutations) was performed to exclude the potential overfitting of this model with satisfactory R^2^ and Q^2^ for each group (R^2^, Q^2^ for Gyeryong class, 0.182, −0.5310; R^2^, Q^2^ for Hongcheon class, 0.149, −0.549; R^2^, Q^2^ for Muju class, 0.098, −0.573; and R^2^, Q^2^ for Samcheonpo class, 0.15, −0.535, respectively; Figure 7). As shown in Figure 8a, the PLS-DA score plot drawn using the first two predictive components pointed out an excellent discrimination of the LAE considering the four geographical origins investigated. Furthermore, the correct classification rate of 100% was achieved, which indicated that 100% of the samples of the prediction set were correctly classified based on the locations of the samples. Receiver operating characteristic (ROC) curves were also analyzed for the tested classes of geographical origin (Gyeryong, Hongcheon, Muju, and Samcheonpo). As a result, the area under the ROC (AUC) was acquired for all the tested groups, indicating the excellent performance of this classification model.

The PLS-DA model was established for the geographical authentication of LAE and was subsequently examined to identify differential secondary metabolites, so-called markers, that are responsible for the discrimination between the investigated categories. The VIP approach, which shows the contribution of a variable to the established PLS-DA model, was applied for that purpose, and features with VIP scores > 1.5 were selected as the potential markers for LAE authentication. As a result, Figure 8b displayed the initially detected features in LAE and peak 8 (quercitrin) shows the potential to discriminate the samples from different cities. This compound has been reported to have several biological effects, including anti-inflammatory and antioxidant activities, and may contribute to the medicinal properties of *A. elata*-based products.

The identification of potential markers for the geographical authentication of LAE has important implications for the quality control and authentication of LAE-based products. The use of natural products is widespread in traditional medicine, but quality control is often lacking, and the authenticity and consistency of these products can be difficult to ensure. The identification of potential markers for the discrimination of LAE samples based on their chemical compositions can help to ensure the authenticity and consistency of LAE-based products and improve their quality control.

## 3. Materials and Methods

### 3.1. Plant Materials

A total of 21 samples of *A. elata* leaves were collected from various regions in South Korea, including Gyeryong, Hongcheon, Muju, and Samcheonpo. Specifically, samples 1 to 6 were harvested from Samcheonpo on 10 August 2021. Samples S7 to S9 were harvested from Muju on 9 August 2021. Samples S10 to S13 were harvested from Hongcheon on 9 August 2021. And samples S14 to S21 were harvested from Gyeryong on 9 August 2021. To prepare the extracts, we used 100 g of plant material from each origin. The collection was carried out by Professor Jong Seong Kang of the College of Pharmacy at Chungnam National University. Professor Jong Seong Kang identified the samples, and their collection information, including the date, location, quantity, longitude, and latitude, is listed in Table S1. Vouchers for each sample were deposited in the pharmaceutical analysis laboratory at the College of Pharmacy with the code number LAE1-21.

### 3.2. Chemical and Reagents

To ensure high-quality and reliable results, the following materials were used in the study. Ultrapure water with a resistivity of 18.2 MΩ cm was obtained from the Mili-Q water purification system (Milford, MA, USA). Acetonitrile, used for HPLC measurements, was of LC-MS grade and purchased from Burdick & Jackson (Muskegon, MI, USA). Formic acid was used as a mobile phase modifier and was of mass-spectrometry grade and obtained from Sigma-Aldrich (Darmstadt, Germany).

EndoFectin™ Max Transfection Reagent (EFM1004) was purchased from GeneCopoeia, located in Rockville, MD, USA. DEAE-Dextran hydrochloride (#D9885) and Ang II (#A9525) were purchased from Sigma-Aldrich in St. Louis, MO, USA. FMK (#Axon 1848) was purchased from Axon Medchem in VA, USA. Finally, the reagent of 3-(4,5-dimethylthiazol-2-yl)-2,5-diphenyltetrazolium bromide (MTT) was bought from Sigma-Aldrich Chemie, located in St. Louis, MO, USA.

### 3.3. Preparation of Leaf Extracts

The LAE sample was dried in an LTO-Do-150S oven (Labtech-one, Namyangju-si, Republic of Korea) at 50 °C for 48 h in order to maintain a moisture content below 13%. Subsequently, the dried leaves were crushed and 1 g was added to 10 mL of 70% (*v*/*v*) ethanol. To facilitate extraction, the mixture was subjected to ultrasonication at 40 kHz and 50 °C for 60 min using a Mujigae ultrasonic bath machine (Seoul, Republic of Korea). Following extraction, centrifugation was conducted for 5 min at 4000 rpm. The resulting supernatant was clarified and filtered through a 0.22 μm membrane syringe filter prior to analysis using the HPLC system. All solutions were stored at 4 °C until analysis.

### 3.4. HPLC–DAD–ESI–MS/MS Analysis

The bioactive components (triterpenoid saponin) present in LAE were identified using a Prominence TM high-performance liquid chromatography (HPLC) system equipped with an electrospray ionization (ESI) source and a triple quadrupole mass spectrometer (Shimadzu, Kyoto, Japan). The RStech HECTOR m C18 column (250 × 4.6 mm, 5 μm; Daejeon, Republic of Korea) was employed for the chromatographic separation, with the column temperature set at 30 °C. The mobile phase was a mixture of water (A, containing 0.1% formic acid) and acetonitrile (B, containing 0.1% formic acid), with a flow rate of 0.5 mL/min. The gradient condition was optimized as follows: 10%–40% B for 0–100 min. The optimal ionization parameters were set as follows: drying gas 15 L/min; desolvation line temperature, 250 °C; heat block temperature, 400 °C; nebulizing gas 3 L/min; and scan range from *m*/*z* 100–1500 Da.

### 3.5. Quality Control of LAE

For HPLC analysis of the LAE samples, a Shimadzu HPLC system equipped with UV was used. The RStech HECTOR m C18 column (250 × 4.6 mm, 5 μm; Daejeon, Republic of Korea) was used at a flow rate of 1 mL/min and the mobile phase was the same as that used for LC–MS analysis. The gradient condition was optimized as follows: 10%–40% B for 0–50 min. Detection of multiple bioactive components was achieved by monitoring at a detection wavelength of 254 nm.

To analyze the triterpenoids present in the LAE samples, an HPLC–UV system (Shimzadu LC-20 A series system, Shimadzu, Kyoto, Japan) coupled with an SEDEX-LTE 80 ELSD was used. The optimal conditions were a carrier N_2_ gas flow rate of 3.5 L/min and a drift tube temperature of 50 °C. A solvent A combination of water and 0.1% formic acid (*v*/*v*) and solvent B (acetonitrile) were employed for gradient elution using the following program: 27–28.5% B for 0–25 min and 28.5–70% B for 25–70 min. Column temperature and flow rate were set to 30 °C and 1 mL/min, respectively.

### 3.6. Cell Culture

The human umbilical vein endothelial cells (HUVECs) were isolated from collagenase-treated umbilical cord veins. Next, HUVECs were collected in M200 medium containing LSGS (Cascade Biologics, Inc., Portland, OR, USA) and 2% fetal bovine serum (FBS; Atlanta Biologicals, Inc., Lawrenceville, GA, USA). The cells were then seeded in Petri dishes coated with 0.2% gelatin type A (#901771; MP Biomedicals, Santa Ana, CA, USA) and cultured in Endothelial Cell Medium (ECM, #1001, ScienCell, Carlsbad, CA, USA) supplemented with 5% (*v*/*v*) FBS (#0025, ScienCell), Endothelial Cell Growth Supplement (ECGS, #1052, ScienCell, Carlsbad, CA) (5 mL), and penicillin/streptomycin (P/S, #0503, ScienCell, Carlsbad, CA, USA) (5 mL). Finally, all HUVECs were maintained in culture at 37 °C in a 5% CO_2_-humidified atmosphere.

### 3.7. Cell Viability Assay

The viability of cultured HUVECs was assessed using the MTT assay. Briefly, HUVECs were seeded at a density of 1 × 10^4^ cells per well in a 96-well plate and allowed to attach for 24 h prior to serum starvation using fresh FBS-free medium for an additional 24 h. Cells were then exposed to increasing concentrations (0, 1, 2.5, 5, and 10 μg/mL) of the test extracts, which were prepared by sample 1 harvested from Samcheonpo for another 24 h. Following exposure, each well was treated with a 12 mM MTT solution in FBS-free Dulbecco’s modified Eagle medium (DMEM), and incubated for 2 h at 37 °C and 5% CO_2_. Next, the medium was aspirated, and cell–formazan precipitates were solubilized with DMSO and quantified spectrophotometrically at 540 nm using a microplate reader (TECAN, Männedorf, Switzerland).

### 3.8. Western Blotting

To investigate the effects of LAE extracts on lipopolysaccharide (LPS)-induced inflammation, HUVECs were treated with either vehicle or LAE extracts (5 μg/mL) followed by LPS (200 ng/mL). The following antibodies were purchased from various suppliers: rabbit anti-phospho-Akt ser473 (#9271), rabbit anti-Akt (#9272), rabbit anti-phospho-ERK1/2(#4370), rabbit anti-ERK1/2 (#4695), and rabbit anti-phospho-p90RSK (Ser380) (#11989) were purchased from Cell Signaling Technology in Danvers, MA, USA; mouse anti-ICAM-1 (#sc-8439), mouse VCAM-1 (#sc-13160), and mouse anti-NF-kB p65 (#sc-514451) were obtained from Santa Cruz Technology, Inc. (Santa Cruz, CA, USA). Subsequently, cells were washed with phosphate-buffered saline (PBS), and 2X SDS lysis buffer (composed of 1 M Tris-HCl pH 7.4, 10% SDS, 25% glycerol, 5% 2-mercaptoethanol, and 1% bromophenol blue) was used to lyse the cells. Protein extracts were then resolved using sodium dodecyl sulfate–polyacrylamide gel electrophoresis followed by detection with enhanced chemiluminescence reagents (Amersham Pharmacia Biotech, Piscataway, NJ, USA) as per the manufacturer’s instructions. Western blotting was used to quantify protein expression levels with each specific antibody matched to the protein of interest.

### 3.9. Statistical Analysis

Multivariate statistical analyses were conducted using MetaboAnalyst 5.0, a free web tool that provides a platform for raw data processing [75]. The original HPLC file underwent various processing steps such as peak detection, data deconvolution, peak area refinement after background filtering, a normalization of missing values, and multivariate data analysis. The peak area dataset was normalized by sum and transformed by logarithm and Pareto scaling (mean-centered and divided by the square root of the standard deviation of each variable). SIMCA version 14.1 software (Umetrics, Umeå, Sweden) was used for unsupervised principal component analysis (PCA) and supervised partial-least-squares discriminate analysis (PLS-DA). To detect any potential outliers, a preliminary assessment of differences between sample groups was carried out using PCA alongside Hotelling’s T^2^ and distance-to-model (DModX) tests. Subsequently, PLS-DA was performed to improve clustering and sample discrimination based on fingerprint variations corresponding to the geographical origin. The models’ quality was assessed by examining the goodness-of-fit parameter (R^2^X), the proportion of variance that the model explains (R^2^Y), and the model’s predictive ability (Q^2^) [76]. A Q^2^ threshold of more than 0.5 was considered good predictability, while R^2^Y and Q^2^ values close to 1 indicated satisfactory model performance [77,78]. The PLS-DA model was validated using default SIMCA 7-fold cross-validation (CV) and permutation tests (200 permutations) upon obtaining a CV-ANOVA *p*-value threshold for significance less than 0.05 [45].

To select markers with high discrimination potential in the predictive model (VIP score > 1.5), the variable importance in projection (VIP) method was employed. These markers were subsequently putatively identified. Analytical data for the selected VIP markers were input into the MetaboAnalyst 5.0 platform (http://www.metaboanalyst.ca/, accessed on 23 June 2023) to obtain box plots showing the differential component peak area based on investigated categories and *p*-values of marker compounds. Univariate analysis using two-sample *t*-tests was used to process markers, with a *p*-value threshold of 0.05, indicating a significant variable for sample discrimination. To compare peak area changes between different origins, log fold-changes (Log FCs) values obtained from fold change analysis were also considered.

## 4. Conclusions

In conclusion, the utilization of the non-medicinal parts of plants or organisms plays a crucial role in resource management and sustainability. By tapping into the potential of these parts, we can maximize resource utilization and minimize waste. Non-medicinal parts often contain valuable components such as fibers, oils, or bioactive compounds that can be utilized for various purposes. This not only promotes a more holistic approach to resource utilization, but also reduces our dependence on limited resources. Additionally, it supports the development of environmentally friendly practices by reducing the overall environmental impact associated with resource extraction. The study of wasted LAE is a topic of increasing importance in environmental and economic fields. This study investigated the anti-inflammatory effects of LAE extract on HUVECs, specifically by focusing on its effect on endothelial cell protective activity and the chemical constituents of the extract. The extract was found to attenuate PI3K/Akt and MAPK signaling pathways, both of which are involved in the production of pro-inflammatory cytokines and are known to exacerbate inflammation in cardiovascular diseases (CVDs). The study also identified specific triterpenoid saponins and potential markers for the geographical authentication of LAE, which have important implications for the quality control and authentication of *A. elata*-based products. The appropriate harvest season and cultivated city must be determined by comparing the content differences between chemical components, and the use of LAE as a treatment material for CVDs can offer a cost-effective, sustainable, and natural source of medicinal plant extracts. Further research is needed to explore the potential synergistic effects of the identified constituents, elucidate their bioavailability and pharmacokinetics, and evaluate the clinical efficacy of LAE-based products. Nonetheless, the identification of specific triterpenoid saponins may pave the way for the development of novel natural anti-inflammatory agents for various inflammatory disorders. The study provides important insights into the potential therapeutic benefits of LAE extract for CVD, while also contributing to the preservation of natural resources and reducing waste.

## Data Availability

Data are contained within the article and Supplementary Materials.

## Supplementary Materials

The following supporting information can be downloaded at: https://www.mdpi.com/article/10.3390/molecules28155907/s1, Figure S1: HPLC–UV chromatograms of *A. elata* leaves harvest from different seasons determined at 254 nm. Peak 1: chlorogenic acid; peak 5, rutin; peak 6: hyperoside, peak 7: isochlorogenic acid A; peak 8: quercitrin, peak 9: isochlorogenic C; other peaks were unknown; Figure S2: (a) Hotelling’s T^2^ plot shows how far each sample is from the model center using confidence levels of 95% and 99% for moderate and strong outliers; (b) DModX plot displaying the deviations between the observed data and the PCA model. The samples with DModX values larger than the DCrit (red line) are regarded as the outliers. Table S1: Source of 21 batches *A. elata* leaves samples.

## References

[1] Levy R.I., Moskowitz J. (1982). Cardiovascular research: Decades of progress, a decade of promise. Science.

[2] Zhou Q., Han X., Li R., Zhao W., Bai B., Yan C., Dong X. (2018). Anti-atherosclerosis of oligomeric proanthocyanidins from *Rhodiola rosea* on rat model via hypolipemic, antioxidant, anti-inflammatory activities together with regulation of endothelial function. Phytomedicine.

[3] Kahleova H., Levin S., Barnard N.D. (2018). Vegetarian dietary patterns and cardiovascular disease. Prog. Cardiovasc. Dis..

[4] Incalza M.A., D’Oria R., Natalicchio A., Perrini S., Laviola L., Giorgino F. (2018). Oxidative stress and reactive oxygen species in endothelial dysfunction associated with cardiovascular and metabolic diseases. Vasc. Pharmacol..

[5] Zhang Y., Zhou H., Wu W., Shi C., Hu S., Yin T., Ma Q., Han T., Zhang Y., Tian F. (2016). Liraglutide protects cardiac microvascular endothelial cells against hypoxia/reoxygenation injury through the suppression of the SR-Ca^2+^–XO–ROS axis via activation of the GLP-1R/PI3K/Akt/survivin pathways. Free Radic. Biol. Med..

[6] Libby P., Ridker P.M., Hansson G.K., Leducq Transatlantic Network on Atherothrombosis (2009). Inflammation in atherosclerosis: From pathophysiology to practice. J. Am. Coll. Cardiol..

[7] Kaperonis E., Liapis C., Kakisis J., Dimitroulis D., Papavassiliou V. (2006). Inflammation and atherosclerosis. Eur. J. Vasc. Endovasc. Surg..

[8] Estruch R., Sacanella E., Badia E., Antúnez E., Nicolás J.M., Fernández-Solá J., Rotilio D., De Gaetano G., Rubin E., Urbano-Márquez A. (2004). Different effects of red wine and gin consumption on inflammatory biomarkers of atherosclerosis: A prospective randomized crossover trial: Effects of wine on inflammatory markers. Atherosclerosis.

[9] Palmefors H., DuttaRoy S., Rundqvist B., Börjesson M. (2014). The effect of physical activity or exercise on key biomarkers in atherosclerosis—A systematic review. Atherosclerosis.

[10] Hein T.W., Singh U., Vasquez-Vivar J., Devaraj S., Kuo L., Jialal I. (2009). Human C-reactive protein induces endothelial dysfunction and uncoupling of eNOS in vivo. Atherosclerosis.

[11] Teoh H., Quan A., Lovren F., Wang G., Tirgari S., Szmitko P.E., Szalai A.J., Ward M.E., Verma S. (2008). Impaired endothelial function in C-reactive protein overexpressing mice. Atherosclerosis.

[12] Yosimichi G., Nakanishi T., Nishida T., Hattori T., Takano-Yamamoto T., Takigawa M. (2001). CTGF/Hcs24 induces chondrocyte differentiation through a p38 mitogen-activated protein kinase (p38MAPK), and proliferation through a p44/42 MAPK/extracellular-signal regulated kinase (ERK). Eur. J. Biochem..

[13] Ijomone O.M., Iroegbu J.D., Aschner M., Bornhorst J. (2021). Impact of environmental toxicants on p38-and ERK-MAPK signaling pathways in the central nervous system. Neurotoxicology.

[14] Lim H.-S., Kim Y.J., Kim B.-Y., Jeong S.-J. (2019). Bakuchiol suppresses inflammatory responses via the downregulation of the p38 MAPK/ERK signaling pathway. Int. J. Mol. Sci..

[15] Mudaliar H., Rayner B., Billah M., Kapoor N., Lay W., Dona A., Bhindi R. (2017). Remote ischemic preconditioning attenuates EGR-1 expression following myocardial ischemia reperfusion injury through activation of the JAK-STAT pathway. Int. J. Cardiol..

[16] Bolli R., Dawn B., Xuan Y.-T. (2003). Role of the JAK–STAT pathway in protection against myocardial ischemia/reperfusion injury. Trends Cardiovasc. Med..

[17] Xi S., Zhou G., Zhang X., Zhang W., Cai L., Zhao C. (2009). Protective effect of total aralosides of *Aralia elata* (Miq) Seem (TASAES) against diabetic cardiomyopathy in rats during the early stage, and possible mechanisms. Exp. Mol. Med..

[18] Wang M., Xu X., Xu H., Wen F., Zhang X., Sun H., Yao F., Sun G., Sun X. (2014). Effect of the total saponins of *Aralia elata* (Miq) Seem on cardiac contractile function and intracellular calcium cycling regulation. J. Ethnopharmacol..

[19] Hwang K.-A., Hwang Y.-J., Kim G.R., Choe J.-S. (2015). Extracts from *Aralia elata* (Miq) Seem alleviate hepatosteatosis via improving hepatic insulin sensitivity. BMC Complement. Altern. Med..

[20] Zhang J., Wang H., Xue Y., Zheng Q. (2013). Cardioprotective and antioxidant activities of a polysaccharide from the root bark of *Aralia elata* (Miq.) Seem. Carbohydr. Polym..

[21] Luo Y., Dong X., Yu Y., Sun G., Sun X. (2015). Total aralosides of *Aralia elata* (Miq) Seem (TASAES) ameliorate nonalcoholic steatohepatitis by modulating IRE1α-mediated JNK and NF-κB pathways in ApoE–/–mice. J. Ethnopharmacol..

[22] Tian Y.-q., Zhao H.-t., Zhang X.-l., Zhang W.-t., Liu X.-c., Gao S.-h. (2020). Comparison of different extraction techniques and optimization of the microwave-assisted extraction of saponins from *Aralia elata* (Miq.) Seem fruits and rachises. Chem. Pap..

[23] Luo Y., Lu S., Ai Q., Zhou P., Qin M., Sun G., Sun X. (2019). SIRT1/AMPK and Akt/eNOS signaling pathways are involved in endothelial protection of total aralosides of *Aralia elata* (Miq) Seem against high-fat diet-induced atherosclerosis in ApoE−/− mice. Phytother. Res..

[24] Zhou P., Xie W., Luo Y., Lu S., Dai Z., Wang R., Sun G., Sun X. (2018). Protective effects of total saponins of *Aralia elata* (Miq.) on endothelial cell injury induced by TNF-α via modulation of the PI3K/Akt and NF-κB signalling pathways. Int. J. Mol. Sci..

[25] Zhang Y., Wang W., He H., Song X.-y., Yao G.-d., Song S.-j. (2018). Triterpene saponins with neuroprotective effects from a wild vegetable *Aralia elata*. J. Funct. Foods.

[26] Wang W., Yao G.-D., Shang X.-Y., Gao J.-C., Zhang Y., Song S.-J. (2018). Eclalbasaponin I from *Aralia elata* (Miq.) Seem. reduces oxidative stress-induced neural cell death by autophagy activation. Biomed. Pharmacother..

[27] Wang Z., Wu Q., Meng Y., Sun Y., Wang Q., Yang C., Wang Q., Yang B., Kuang H. (2015). Determination and pharmacokinetic study of two triterpenoid saponins in rat plasma after oral administration of the extract of *Aralia elata* leaves by UHPLC–ESI–MS/MS. J. Chromatogr. B.

[28] Chen L., Tao Y., Jiang Y. (2015). Apelin activates the expression of inflammatory cytokines in microglial BV2 cells via PI-3K/Akt and MEK/Erk pathways. Sci. China Life Sci..

[29] Gao H.-L., Yu X.-J., Feng Y.-Q., Yang Y., Hu H.-B., Zhao Y.-Y., Zhang J.-H., Liu K.-L., Zhang Y., Fu L.-Y. (2023). Luteolin Attenuates Hypertension via Inhibiting NF-κB-Mediated Inflammation and PI3K/Akt Signaling Pathway in the Hypothalamic Paraventricular Nucleus. Nutrients.

[30] An W., Yang J., Ao Y. (2006). Metallothionein mediates cardioprotection of isoliquiritigenin against ischemia-reperfusion through JAK2/STAT3 activation. Acta Pharmacol. Sin..

[31] Burysek L., Syrovets T., Simmet T. (2002). The serine protease plasmin triggers expression of MCP-1 and CD40 in human primary monocytes via activation of p38 MAPK and janus kinase (JAK)/STAT signaling pathways. J. Biol. Chem..

[32] Biesaga M., Pyrzynska K. (2009). Liquid chromatography/tandem mass spectrometry studies of the phenolic compounds in honey. J. Chromatogr. A.

[33] Zhang Y., Peng Y., Li L., Zhao L., Hu Y., Hu C., Song S. (2013). Studies on cytotoxic triterpene saponins from the leaves of *Aralia elata*. Food Chem..

[34] Kuang H.X., Wang Z.B., Wang Q.H., Yang B.Y., Xiao H.B., Okada Y., Okuyama T. (2013). Triterpene glucosides from the leaves of *Aralia elata* and their cytotoxic activities. Chem. Biodivers..

[35] Li F., He X., Niu W., Feng Y., Bian J., Xiao H. (2015). Acute and sub-chronic toxicity study of the ethanol extract from leaves of *Aralia elata* in rats. J. Ethnopharmacol..

[36] Timalsina D., Devkota H.P. (2021). *Eclipta prostrata* (L.) L.(Asteraceae): Ethnomedicinal uses, chemical constituents, and biological activities. Biomolecules.

[37] Mroczek A. (2015). Phytochemistry and bioactivity of triterpene saponins from Amaranthaceae family. Phytochem. Rev..

[38] Xu Y., Liu J., Zeng Y., Jin S., Liu W., Li Z., Qin X., Bai Y. (2022). Traditional uses, phytochemistry, pharmacology, toxicity and quality control of medicinal genus Aralia: A review. J. Ethnopharmacol..

[39] Han F., Liang J., Yang B.-Y., Kuang H.-X., Xia Y.-G. (2021). Identification and comparison of triterpene saponins in *Aralia elata* leaves and buds by the energy-resolved MSAll technique on a liquid chromatography/quadrupole time-of-flight mass spectrometry. J. Pharm. Biomed. Anal..

[40] Qi M., Hua X., Peng X., Yan X., Lin J. (2018). Comparison of chemical composition in the buds of *Aralia elata* from different geographical origins of China. R. Soc. Open Sci..

[41] Petrochenko A.A., Orlova A., Frolova N., Serebryakov E.B., Soboleva A., Flisyuk E.V., Frolov A., Shikov A.N. (2023). Natural Deep Eutectic Solvents for the Extraction of Triterpene Saponins from *Aralia elata* var. *mandshurica* (Rupr. & Maxim.) J. Wen. Molecules.

[42] Guo M., Zhang L., Liu Z. (2009). Analysis of saponins from leaves of *Aralia elata* by liquid chromatography and multi-stage tandem mass spectrometry. Anal. Sci..

[43] Wang Y., Zhang H., Ri H.C., An Z., Wang X., Zhou J.-N., Zheng D., Wu H., Wang P., Yang J. (2022). Deletion and tandem duplications of biosynthetic genes drive the diversity of triterpenoids in *Aralia elata*. Nat. Commun..

[44] Gao D., Vinh L.B., Cho C.W., Cho K.W., Kim Y.H., Kang J.S. (2020). Discrimination and quality evaluation of fifteen components in *Stauntonia hexaphylla* leaves from different harvest time by HPLC–PDA–ESI–MS/MS and ELSD coupled with multivariate statistical analysis and anti-inflammatory activity evaluation. Appl. Biol. Chem..

[45] Gao D., Cho C.-W., Kim J.-H., Bao H., Kim H.-M., Li X., Kang J.-S. (2022). Phenolic Profile and Fingerprint Analysis of *Akebia quinata* Leaves Extract with Endothelial Protective Activity. Molecules.

[46] Shikov A.N., Pozharitskaya O.N., Makarov V.G. (2016). *Aralia elata* var. *mandshurica* (Rupr. & Maxim.) J. Wen: An overview of pharmacological studies. Phytomedicine.

[47] Solar A., Colarič M., Usenik V., Stampar F. (2006). Seasonal variations of selected flavonoids, phenolic acids and quinones in annual shoots of common walnut (*Juglans regia* L.). Plant Sci..

[48] Trigui M., Gasmi L., Zouari I., Tounsi S. (2013). Seasonal variation in phenolic composition, antibacterial and antioxidant activities of *Ulva rigida* (Chlorophyta) and assessment of antiacetylcholinesterase potential. J. Appl. Phycol..

[49] Souhila T., Fatma Zohra B., Tahar H.S. (2019). Identification and quantification of phenolic compounds of *Artemisia herba-alba* at three harvest time by HPLC–ESI–Q-TOF–MS. Int. J. Food Prop..

[50] Formato M., Scharenberg F., Pacifico S., Zidorn C. (2022). Seasonal variations in phenolic natural products in *Fagus sylvatica* (European beech) leaves. Phytochemistry.

[51] Fang R., Redfern S.P., Kirkup D., Porter E.A., Kite G.C., Terry L.A., Berry M.J., Simmonds M.S. (2017). Variation of theanine, phenolic, and methylxanthine compounds in 21 cultivars of *Camellia sinensis* harvested in different seasons. Food Chem..

[52] Sartor T., Xavier V., Falcão M., Mondin C., Dos Santos M., Cassel E., Astarita L., Santarém E. (2013). Seasonal changes in phenolic compounds and in the biological activities of *Baccharis dentata* (Vell.) GM Barroso. Ind. Crops Prod..

[53] Ciriello M., Formisano L., El-Nakhel C., Kyriacou M.C., Soteriou G.A., Pizzolongo F., Romano R., De Pascale S., Rouphael Y. (2021). Genotype and successive harvests interaction affects phenolic acids and aroma profile of genovese basil for pesto sauce production. Foods.

[54] Xin Q., Liu B., Sun J., Fan X., Li X., Jiang L., Hao G., Pei H., Zhou X. (2022). Heat shock treatment promoted callus formation on postharvest sweet potato by adjusting active oxygen and phenylpropanoid metabolism. Agriculture.

[55] Lim D.W., Han T., Jung J., Song Y., Um M.Y., Yoon M., Kim Y.T., Cho S., Kim I.H., Han D. (2018). Chlorogenic Acid from Hawthorn berry (*Crataegus pinnatifida* fruit) prevents stress hormone-induced depressive behavior, through monoamine oxidase b-reactive oxygen species signaling in hippocampal astrocytes of mice. Mol. Nutr. Food Res..

[56] Shimomura M., Yoshida H., Fujiuchi N., Ariizumi T., Ezura H., Fukuda N. (2020). Continuous blue lighting and elevated carbon dioxide concentration rapidly increase chlorogenic acid content in young lettuce plants. Sci. Hortic..

[57] Brazaitytė A., Vaštakaitė-Kairienė V., Sutulienė R., Rasiukevičiūtė N., Viršilė A., Miliauskienė J., Laužikė K., Valiuškaitė A., Dėnė L., Chrapačienė S. (2022). Phenolic compounds content evaluation of lettuce grown under short-term preharvest daytime or nighttime supplemental LEDs. Plants.

[58] Wargent J., Nelson B., McGhie T., Barnes P. (2015). Acclimation to UV-B radiation and visible light in L actuca sativa involves up-regulation of photosynthetic performance and orchestration of metabolome-wide responses. Plant Cell Environ..

[59] Aerts R.J., Baumann T.W. (1994). Distribution and utilization of chlorogenic acid in Coffea seedlings. J. Exp. Bot..

[60] Jan R., Asaf S., Numan M., Lubna, Kim K.-M. (2021). Plant secondary metabolite biosynthesis and transcriptional regulation in response to biotic and abiotic stress conditions. Agronomy.

[61] Soliman M.H., Abdulmajeed A.M., Alhaithloul H., Alharbi B.M., El-Esawi M.A., Hasanuzzaman M., Elkelish A. (2020). Saponin biopriming positively stimulates antioxidants defense, osmolytes metabolism and ionic status to confer salt stress tolerance in soybean. Acta Physiol. Plant..

[62] Lazo-Vélez M.A., Guajardo-Flores D., Mata-Ramírez D., Gutiérrez-Uribe J.A., Serna-Saldivar S.O. (2016). Characterization and quantitation of triterpenoid saponins in raw and sprouted *Chenopodium berlandieri* spp.(Huauzontle) grains subjected to germination with or without selenium stress conditions. J. Food Sci..

[63] Falcão E.L., Muniz B.C., Bastos Filho C.J.A., Kapoor R., da Silva F.S.B. (2023). Soil microbial respiration and pH modulated by arbuscular mycorrhizal fungi influence the biosynthesis of health-promoting compounds in *Anadenanthera colubrina* (Vell.) Brenan. Rhizosphere.

[64] Weng Y., Yu L., Cui J., Zhu Y.-R., Guo C., Wei G., Duan J.-L., Yin Y., Guan Y., Wang Y.-H. (2014). Antihyperglycemic, hypolipidemic and antioxidant activities of total saponins extracted from Aralia taibaiensis in experimental type 2 diabetic rats. J. Ethnopharmacol..

[65] Tian Y., Zhang X., Liu H., Gong D., Li X. (2021). Comparison of the nutritional and phytochemical composition and antioxidant activities of *Aralia elata* (Miq.) Seem fruits in Northeast China. Arab. J. Chem..

[66] Hou L., Li S., Tong Z., Yuan X., Xu J., Li J. (2021). Geographical variations in fatty acid and steroid saponin biosynthesis in Dioscorea zingiberensis rhizomes. Ind. Crops Prod..

[67] La Pierre K.J., Blumenthal D.M., Brown C.S., Klein J.A., Smith M.D. (2016). Drivers of variation in aboveground net primary productivity and plant community composition differ across a broad precipitation gradient. Ecosystems.

[68] Toledo M., Poorter L., Peña-Claros M., Alarcón A., Balcázar J., Leaño C., Licona J.C., Llanque O., Vroomans V., Zuidema P. (2011). Climate is a stronger driver of tree and forest growth rates than soil and disturbance. J. Ecol..

[69] Laddomada B., Blanco A., Mita G., D’Amico L., Singh R.P., Ammar K., Crossa J., Guzmán C. (2021). Drought and heat stress impacts on phenolic acids accumulation in durum wheat cultivars. Foods.

[70] Puente-Garza C.A., Meza-Miranda C., Ochoa-Martínez D., García-Lara S. (2017). Effect of in vitro drought stress on phenolic acids, flavonols, saponins, and antioxidant activity in *Agave salmiana*. Plant Physiol. Biochem..

[71] Sarker U., Oba S. (2018). Drought stress enhances nutritional and bioactive compounds, phenolic acids and antioxidant capacity of *Amaranthus leafy* vegetable. BMC Plant Biol..

[72] Dias M.C., Pinto D.C., Figueiredo C., Santos C., Silva A.M. (2021). Phenolic and lipophilic metabolite adjustments in *Olea europaea* (olive) trees during drought stress and recovery. Phytochemistry.

[73] Suganthy N., Devi K.P., Nabavi S.F., Braidy N., Nabavi S.M. (2016). Bioactive effects of quercetin in the central nervous system: Focusing on the mechanisms of actions. Biomed. Pharmacother..

[74] Islam M.S., Quispe C., Hossain R., Islam M.T., Al-Harrasi A., Al-Rawahi A., Martorell M., Mamurova A., Seilkhan A., Altybaeva N. (2021). Neuropharmacological effects of quercetin: A literature-based review. Front. Pharmacol..

[75] Pang Z., Zhou G., Ewald J., Chang L., Hacariz O., Basu N., Xia J. (2022). Using MetaboAnalyst 5.0 for LC–HRMS spectra processing, multi-omics integration and covariate adjustment of global metabolomics data. Nat. Protoc..

[76] Virgiliou C., Kanelis D., Pina A., Gika H., Tananaki C., Zotou A., Theodoridis G. (2020). A targeted approach for studying the effect of sugar bee feeding on the metabolic profile of Royal Jelly. J. Chromatogr. A.

[77] Dahabiyeh L.A., Mansour R.S., Saleh S.S., Kamel G. (2020). Investigating the molecular structure of placenta and plasma in pre-eclampsia by infrared microspectroscopy. J. Pharm. Biomed. Anal..

[78] Yang Y., Zhu H., Chen J., Xie J., Shen S., Deng Y., Zhu J., Yuan H., Jiang Y. (2022). Characterization of the key aroma compounds in black teas with different aroma types by using gas chromatography electronic nose, gas chromatography-ion mobility spectrometry, and odor activity value analysis. LWT.

