# Supplementary material for: Identification and Quantitation of the Bioactive Components in Wasted Aralia elata Leaves Extract with Endothelial Protective Activity
Source: Molecules. 2023 Aug 6;28(15):5907. doi: 10.3390/molecules28155907 (PMC10421206; doi:10.3390/molecules28155907)
Supplement: Supplementary file 1 [file molecules-28-05907-s001.zip › molecules-2528308-supplementary.pdf]

molecules

Supplementary Materials

Identification and Quantitation of the Bioactive Components in wasted Aralia elata leaves extract with Endothelial Protective Activity

Dan Gao [1], Chong-Woon Cho [2], Zemin Yang [1,3], Xiwen Li [1,*] and Jong-Seong Kang [2,*]

[1] Institute of Chinese Materia Medica, China Academy of Chinese Medical Sciences, Beijing 100700, China; dgao@icmm.ac.cn (D.G.), yangzm0615@163.com (Z.M.Y.)
[2] College of Pharmacy, Chungnam National University, Daejeon 34134, Korea; chongw113@cnu.ac.kr (C.W.C)
[3] College of Traditional Chinese Medicine, Yunnan University of Chinese Medicine, Kunming 650500,China

* Correspondence: xwli@icmm.ac.cn (X.W.L.); kangjss@cnu.ac.kr (J.S.K.); Tel.: +86-10-8408-4107(X.W.L.); +82-42-821-5928 (J.S.K.)

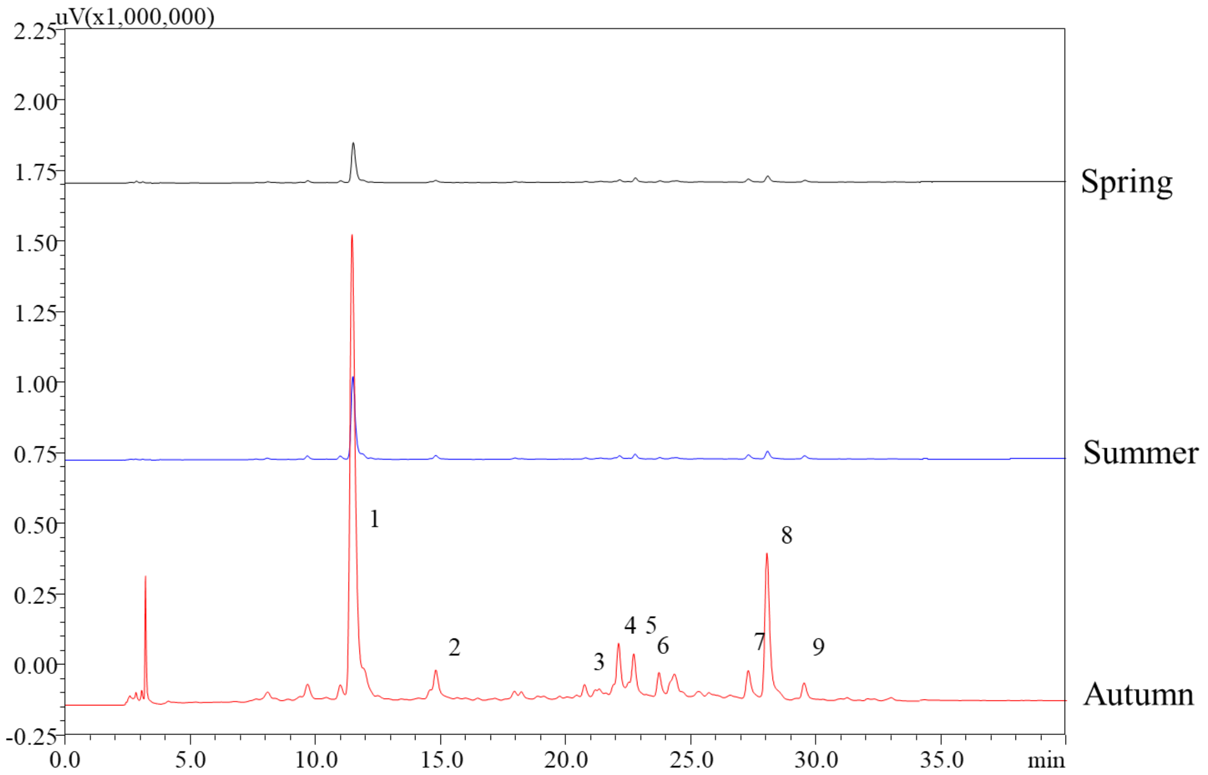

Figure S1. HPLC–UV chromatograms of *A. elata* leaves harvest from different seasons determined at 254 nm. Peak 1: chlorogenic acid; peak 5, rutin; peak 6: hyperoside, peak 7: isochlorogenic acid A; peak 8: quercitrin, peak 9: isochlorogenic C; other peaks were unknown. The samples collected in the spring were dated February 26th, the samples collected in the summer were dated June 20th, and the samples collected in the fall were dated August 21st.

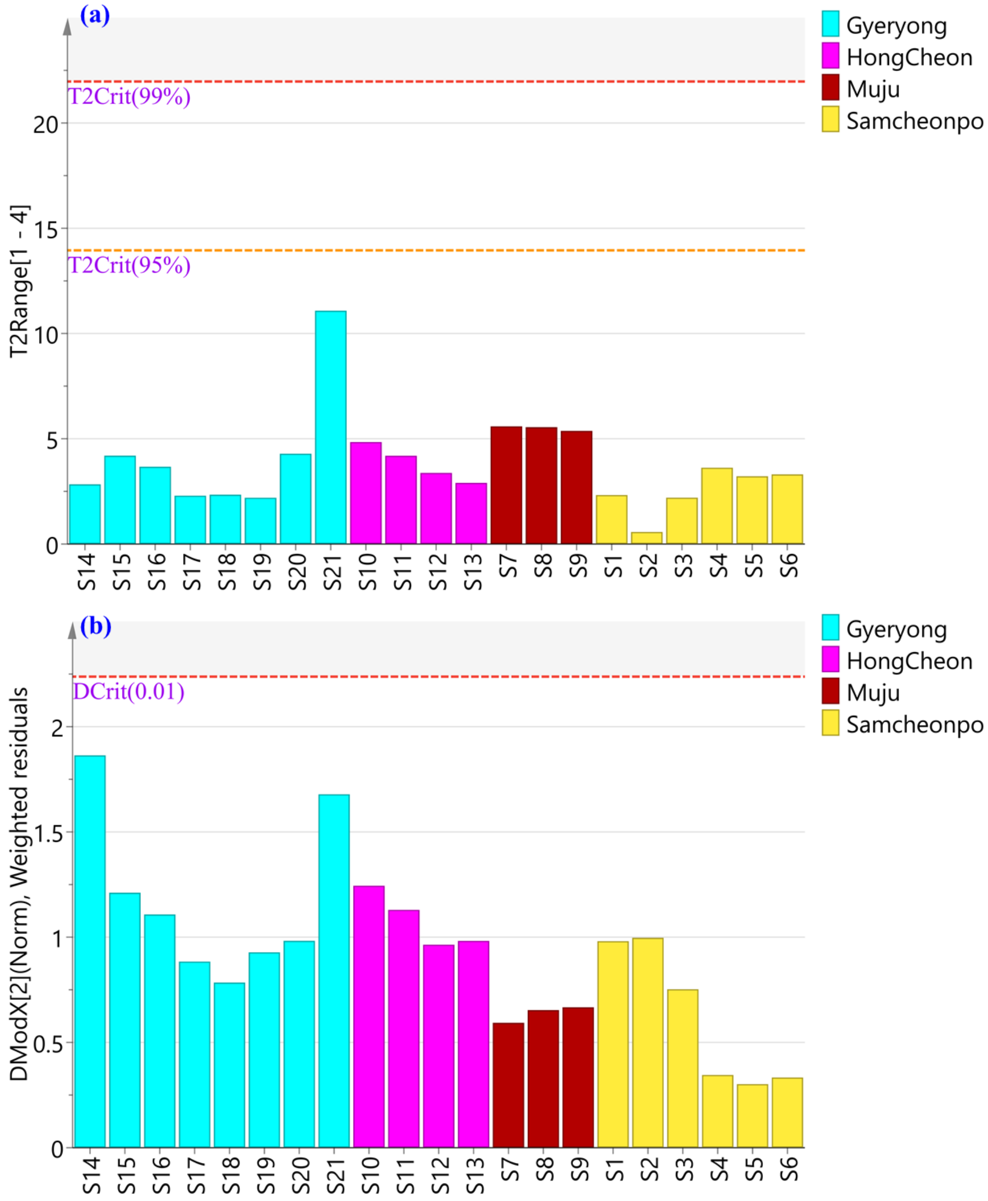

Figure S2. (a) Hotelling's T^2 plot shows how far each sample is from the model center using confidence levels of 95% and 99% for moderate and strong outliers; (b) DModX plot displaying the deviations between the observed data and the PCA model. The samples with DModX values larger than the DCrit (red line) are regarded as the outliers.

Table S1. Source of 21 batches *A. elata* leave samples.

**Code**	**Source**	**Position**	**Collect time**	**Tem. (°C)**	**Humidity (H)**	**Average Annual Rainfall (mm)**
S1	Samcheonpo, Korea	34°91′N, 128°09′E	2021.08.10	15.46	45%	750
S2	Samcheonpo, Korea	34°91′N, 128°09′E	2021.08.10	15.46	45%	750
S3	Samcheonpo, Korea	34°91′N, 128°09′E	2021.08.10	15.46	45%	750
S4	Samcheonpo, Korea	34°91′N, 128°09′E	2021.08.10	15.46	45%	750
S5	Samcheonpo, Korea	34°91′N, 128°09′E	2021.08.10	15.46	45%	750
S6	Samcheonpo, Korea	34°91′N, 128°09′E	2021.08.10	15.46	45%	750
S7	Muju, Korea	36°00′N, 127°66′E	2021.08.09	10.32	50%	1000
S8	Muju, Korea	36°00′N, 127°66′E	2021.08.09	10.32	50%	1000
S9	Muju, Korea	36°00′N, 127°66′E	2021.08.09	10.32	50%	1000
S10	HongCheon, Korea	37°91′N, 127°88′E	2021.09.03	14.68	62%	1500
S11	HongCheon, Korea	37°91′N, 127°88′E	2021.09.03	14.68	62%	1500
S12	HongCheon, Korea	37°91′N, 127°88′E	2021.09.03	14.68	62%	1500
S13	HongCheon, Korea	37°91′N, 127°88′E	2021.09.03	14.68	62%	1500
S14	Gyeryong, Korea	36°16′N, 127°14′E	2021.08.21	19.34	65%	1800
S15	Gyeryong, Korea	36°16′N, 127°14′E	2021.08.21	19.34	65%	1800
S16	Gyeryong, Korea	36°16′N, 127°14′E	2021.08.21	19.34	65%	1800
S17	Gyeryong, Korea	36°16′N, 127°14′E	2021.08.21	19.34	65%	1800
S18	Gyeryong, Korea	36°16′N, 127°14′E	2021.08.21	19.34	65%	1800
S19	Gyeryong, Korea	36°16′N, 127°14′E	2021.08.21	19.34	65%	1800
S20	Gyeryong, Korea	36°16′N, 127°14′E	2021.08.21	19.34	65%	1800
S21	Gyeryong, Korea	36°16′N, 127°14′E	2021.08.21	19.34	65%	1800
